# Mitigating eutrophication may elevate neurotoxic mercury risks in global coastal ecosystems

**DOI:** 10.1016/j.eehl.2025.100176

**Published:** 2025-09-06

**Authors:** Xiangyu Kong, Lufeng Chen, Yongguang Yin, Yong Cai, Yanbin Li

**Affiliations:** a Frontiers Science Center for Deep Ocean Multispheres and Earth System, and Key Laboratory of Marine Chemistry Theory and Technology, Ministry of Education and College of Chemistry and Chemical Engineering, Ocean University of China, Qingdao 266100, China; b Hubei Key Laboratory of Environmental and Health Effects of Persistent Toxic Substances, School of Environment and Health, Jianghan University, Wuhan 430056, China; c Research Center for Eco-Environmental Sciences, Chinese Academy of Sciences, Beijing 100085, China; d Department of Chemistry & Biochemistry, Florida International University, Miami, FL 33199, United States

Coastal eutrophication and mercury (Hg) contamination represent dual planetary boundary threats requiring synergistic mitigation. Coastal eutrophication drives algal blooms, hypoxia, and biodiversity loss while endangering human health [1,2]. In recent years, the United Nations and regional countries have implemented extensive remediation measures, including UN SDG 14.1, the Nutrient Pollution—Global Action Network (2024–2034), and the Baltic Sea Action Plan, to address this issue [3,4]. However, these efforts may inadvertently elevate the risks posed by methylmercury (MeHg)—a potent neurotoxin that bioaccumulates through food webs, threatening both ecosystems and humans [5].

These eutrophication management practices may exacerbate MeHg contamination in coastal systems through three irreversible pathways: (1) modulating MeHg production by altering the cycling of organic matter, sulfur, and iron [6]; (2) inhibiting MeHg degradation driven by intracellular reactive oxygen species within algal cells [7]; and (3) promoting Hg bioaccumulation by suppressing somatic growth dilution and lengthening food chains [8,9]. Although future climate change may introduce some uncertainties to the net effect of these processes [10], the collective influence of these practices points toward an escalation of Hg risk in global coastal ecosystems. This could undermine the effectiveness of the Minamata Convention, which entered into force in 2017 to reduce anthropogenic Hg emissions.

Achieving coastal sustainability requires paradigm-shifting strategies that confront the inseparable nexus between eutrophication alleviation and Hg risk intensification. For policymakers, adopting an integrated, multi-stressor governance framework is imperative. Central to this approach is a dual-reduction strategy—enforcing synchronized source controls across agricultural, industrial, and urban wastewater sectors to simultaneously reduce nutrient and Hg discharges. Additionally, long-term monitoring systems must track key indicators under changing local and climatic conditions. To ensure the sustainability of these measures, technological support for relevant sectors—facilitated through industry-academic (IA) collaboration—should be prioritized, alongside robust stakeholder engagement in oversight processes. Together, these multi-tiered efforts are essential for minimizing Hg-related neurotoxic risks, mitigating eutrophication, and advancing sustainable development.

## CRediT authorship contribution statement

**Xiangyu Kong:** Formal analysis, Funding acquisition, Investigation, Software, Validation, Visualization, Writing – original draft, Writing – review & editing. **Lufeng Chen:** Data curation, Investigation, Methodology. **Yongguang Yin:** Formal analysis, Writing – review & editing. **Yong Cai:** Formal analysis, Writing – review & editing. **Yanbin Li:** Conceptualization, Data curation, Funding acquisition, Methodology, Supervision, Writing – review & editing.

## Declaration of competing interests

The authors declare no competing interests.
